# Insights from Spatial Measures of Intolerance to Identifying Pathogenic Variants in Developmental and Epileptic Encephalopathies

**DOI:** 10.3390/ijms24065114

**Published:** 2023-03-07

**Authors:** Michael Silk, Alex de Sá, Moshe Olshansky, David B. Ascher

**Affiliations:** 1 Systems and Computational Biology, Bio21 Institute, University of Melbourne, Parkville, VIC 3052, Australia; 2 Computational Biology and Clinical Informatics, Baker Heart and Diabetes Institute, Melbourne, VIC 3004, Australia; 3 Baker Department of Cardiometabolic Health, University of Melbourne, Parkville, VIC 3010, Australia; 4 School of Chemistry and Molecular Biosciences, University of Queensland, Brisbane City, QLD 4072, Australia

**Keywords:** developmental and epileptic encephalopathies (DEEs), pathogenic variants, structure-based predictors

## Abstract

Developmental and epileptic encephalopathies (DEEs) are a group of epilepsies with early onset and severe symptoms that sometimes lead to death. Although previous work successfully discovered several genes implicated in disease outcomes, it remains challenging to identify causative mutations within these genes from the background variation present in all individuals due to disease heterogeneity. Nevertheless, our ability to detect possible pathogenic variants has continued to improve as *in silico* predictors of deleteriousness have advanced. We investigate their use in prioritising likely pathogenic variants in epileptic encephalopathy patients’ whole exome sequences. We showed that the inclusion of structure-based predictors of intolerance improved upon previous attempts to demonstrate enrichment within epilepsy genes.

## 1. Introduction

Developmental and epileptic encephalopathies (DEEs) are a class of severe, early-onset epilepsies characterised by hypsarrhythmia detected by electroencephalogram (EEG), infantile spasms, multi-form seizures, cognitive and behavioural deficits, and can sometimes lead to death. Many studies have investigated a likely genetic basis underpinning the disease. Nevertheless, identifying causal variants in patients remains challenging due to the apparent disease heterogeneity [1].

Studies investigating the genetic basis of DEE have primarily focused on *de novo* mutations (DNMs), as, due to the early onset and severity of the disease, few causal variants are disseminated and maintained in the healthy population. 30–50% of infants have causative variants with severe DEE. However, in many cases, multiple DNMs may be identified with no prior history of functional studies or previous observation in epilepsy patients [2]. In order to prioritise candidate DNMs that are likely to be the cause of a patient’s phenotype, *in silico* predictors of deleteriousness have proven to be beneficial. However, their overall predictive accuracy is not generalisable and, therefore, far from ideal.

A previous study [3] aimed to identify genes linked to DEE and to identify causal DNMs in a cohort of 356 patients through a collaboration between two consortia (EuroEPINOMICS and Epi4K/EPGP) [3]. A likelihood analysis revealed an enrichment of DNMs in patients compared with a 411-trio exome sequencing control cohort. Furthermore, they observed while analysing their predictions that 75% of the 429 DNMs identified in patients disrupt synaptic transmission regulation.

Since this analysis, our repertoire of valuable datasets and tools has grown, which can significantly improve our ability to assess a variant’s likelihood of pathogenicity. For example, recent large datasets of population variation, such as gnomAD [4], allow us to filter observed variants in patients into the healthy population. In particular, current measurements of missense intolerance may be highly informative for DNM assessment, where it is likely that pathogenic DNMs cluster within intolerant regions due to their severity [5].

Using recent *in silico* predictors of pathogenicity, we revisit the missense variants in the cohort provided by ClinVar. We perform this exploratory analysis to identify whether there is a significant enrichment of predicted damaging variants in patient samples compared to controls. Within this analysis, we investigate whether these new tools allow us to identify a different set of causative variants or epilepsy-associated genes.

Additionally, we investigate the enrichment of likely pathogenic missense variants within genes associated with DEE within the publicly available Epi25K dataset. Our analysis shows enrichment of these variants within these genes despite including both *de novo* and non-*de novo* variants within the DEE patient whole exome sequencing experiments.

## 2. Results

### 2.1. Pathogenic and Background Missense Variants among Epilepsy Genes

We examined missense variants exome-wide and within 34 genes implicated in epileptic encephalopathies using the Epi4K case-ascertained *de novo* missense variants, Epi25K case-ascertained missense variants, and ClinVar missense variants (accessed 16 June 2020).

Thirty-four key genes implicated in epileptic encephalopathies were selected based on a comprehensive literature review by He et al. (2019) [6]. Of the 34 genes, 14 are components of ion channels (*SCN1A*, *SCN2A*, *SCN8A*, *KCNA1*, *KCNA2*, *KCNB1*, *KCNQ2*, *KCNT1*, *CACNA1A*, *GRIN1*, *GRIN2A*, *GRIN2B*, *GRIN2D*, *HCN1*) and 20 are not (*ATP1A2*, *NTRK2*, *SLC2A1*, *SLC6A1*, *STXBP1*, *FGF12*, *YWHAG*, *DYNC1H1*, *SPTAN1*, *ANKRD11*, *EEF1A2*, *FOXG1*, *NACC1*, *CHD2*, *DNM1*, *DNM1L*, *GNAO1*, *HECW2*, *NEDD4L*, *SYNGAP1*).

We evaluated the distributions of commonly used sequence-based *in silico* predictors of deleteriousness on case-ascertained and control variants both exome-wide and within these 34 genes. Table S1 summarises the control groups. First, we compared 276 case-ascertained confirmed *de novo* variants from the Epi4K consortium dataset with 454 control *de novo* variants not derived from epilepsy patients (control group 1). We then used the second set of 762 control variants for comparison with studies of autism spectrum disorders (control group 2). We annotated all variants using the Variant Effect Predictor (release 101) and the plugin dbNSFP 3.5a, confirming that all are missense variants. We also included 1,082,844 missense variants from the Epi25K consortium dataset’s developmental and epileptic encephalopathies (DEE) analysis group for comparison. We contrasted these with the DiscovEHR missense variants, derived from the general population and presumed to be primarily depleted of pathogenic variation. We considered only variants in the DiscovEHR not present in the Epi25K dataset. 

**Figure 1:**
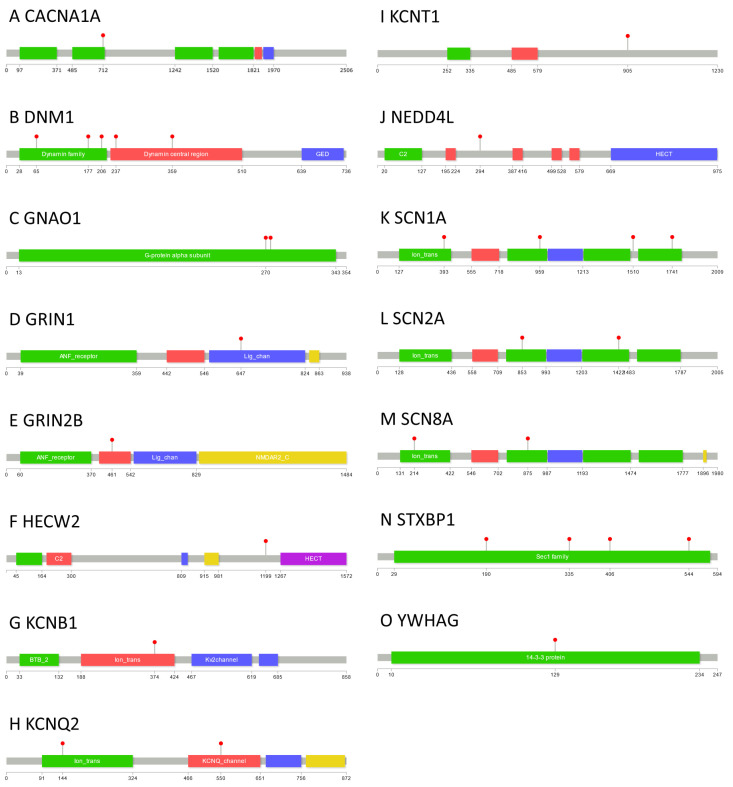
Case-ascertained *de novo* mutation locations within the epilepsy genes. Red lollipops denote the locations of identified variants from the epilepsy cohort in 15 of the 34 target genes. Pfam constitutes the source for the domains.

We were able to locate 29 variants of the 276 case-ascertained *de novo* missense variants within 15 of the 34 genes implicated in DEE (*CACNA1A*: 1, *DNM1*: 5, *GNA01*: 2, *GRIN1*: 1, *GRIN2B*: 1, *HECW2*: 1, *KCNB1*: 1, *KCNQ2*: 2, *KCNT1*: 1, *NEDD4L*: 1, *SCN1A*: 4, *SCN2A*: 2, *SCN8A*: 2, *STXBP1*: 4, *YWHAG*: 1), compared with only 1 control group 1 variant (*NTRK2*: 1) and 3 from control group 2 (*GRIN1*: 1, *SCN1A*: 1, *SLC2A*: 1). These 29 variants are shown in Figure 1 as lollipop plots [7].

We used the ClinVar variant summary dataset to assess the predictive power of a range of *in silico* predictors. We annotated this dataset using the same approach and filtered the missense variants. We performed this procedure to guarantee accurate comparisons to the other variant sets under investigation. In this case, we removed variants with unknown or conflicting significance. While we could not confirm that all variants in this set are related to DEE, the predictive tools used in this study could still provide insights into likely impacts on protein function regardless of the resulting phenotype.

### 2.2. Identifying Missense-Intolerant Regions in Epilepsy Genes

We previously investigated missense intolerance within epilepsy genes and have shown that the Missense Tolerance Ratio (MTR) can be a powerful tool to identify functionally important regions within genes [8]. Since this former study, we recalculated the MTR using additional variation from gnomAD v2, including exomes and genomes, UK Biobank’s 50,000 exomes, and DiscovEHR, increasing the accuracy of scores.

We examined the predictive utility of the updated MTR scores by comparing the number of unique Epi4K variants within the top 25% of most intolerant MTR scores exome-wide, corresponding to an MTR score < 0.78 with control group sets. Of the 276 case-ascertained variants, we observed 91 (33%) within highly intolerant regions compared with 88 (19%) control group 1’s variants and 147 (19%) control group 2’s variants. These results provide signs of other implicated genes harbouring *de novo* variants in disease outcomes.

Considering only variants within the 34 DEE genes, we discovered 21 (72%) case-ascertained variants within intolerant regions, as well as the single (100%) control group 1’s variant and 2 (50%) control group 2’s variants. While we lack sufficient sample sizes to draw direct comparisons between these, it is unsurprising that many control variants are within intolerant regions given how intolerant and conserved these genes are.

Additionally, we examined whether there is an enrichment of Epi25K missense variants within the 34 DEE genes compared with the DiscovEHR control variants after filtering any Epi25K missense variants from the DiscovEHR control variants. Furthermore, we also observed 4656 of the total 10,740 missense variants (43%) within intolerant regions, compared with 6867 of the 17,229 DiscovEHR missense variants (40%).

### 2.3. Investigating Spatial Missense Intolerance within Protein Structures of Epilepsy Genes

We next investigated intolerance within the 34 DEE genes in the context of their tertiary protein structures, utilising experimentally determined structures from the RCSB PDB [9] and homology-modelled structures from SWISS-MODEL [10]. 115 case-ascertained variants, 170 control group 1’s variants and 263 control group 2’s variants had a valid Missense Tolerance Ratio-3D (MTR3D) score for analysis. By defining MTR3D < 0.75 as intolerant, we observe 47 case-ascertained variants (41%), 36 control group 1’s variants (21%) and 58 control group 2 variants (22%). On the other hand, by delineating MTR3D < 0.5 as strongly intolerant, we discovered 22 (19%) case-ascertained variants, 7 control group 1 (4%) and 14 control group 2’s missense variants (5%). Thus, we observed significant enrichment of *de novo* mutations within intolerant regions in the epilepsy analysis group compared with both control groups across all genes with observed DNMs, possibly suggesting other genes implicated in disease outcomes.

Specific to the 34 DEE genes, we could derive MTR3D scores for only 17 case-ascertained variants. The MTR3D score for 16 of the 17 was below 0.75, and 10 of these 16 had an MTR3D score below 0.5, denoting strong intolerance.

Additionally, we utilised the Missense Tolerance Ratio consensus (MTRX), a combined measure of intolerance built through a Random Forest approach using the MTR v1 (41 codons), MTR v2 (21 codons), MTR3D, and residue solvent accessibility (RSA). MTRX scores were available for 92 case-ascertained variants, 151 in control group 1, and 238 in control group 2. We considered MTRX scores approaching 1 more likely to be deleterious. Using a cutoff of 0.75, we found 38 cases-ascertained (41%), 28 control group 1 variants (19%), and 60 control group 2 variants (25%), within these intolerant regions. We could locate 12 case ascertained variants within the 34 DEE genes with a valid MTRX score, 11 of which were within intolerant regions, with an overall mean MTRX score of 0.93.

Next, we calculated conservation for all genes and compared this with the MTR estimates. With all *de novo* variants, we observed a Pearson’s correlation of 0.03 (*p* < 0.58), indicating a low correlation between these scores. However, for the 34 DEE genes, we observed a Pearson’s correlation of 0.34 (*p* < 0.001). These statistics indicate a moderate correlation between these scores within the 34 DEE genes.

### 2.4. Evaluating the Predictive Utility of In-Silico Predictors of Deleteriousness

*In silico* predictors of deleteriousness are of high value in prioritising likely deleterious variants from among background variants when diagnosing rare diseases. Hence, to assess their efficacy in epilepsy, we compared the predictions of several scores obtained through dbNSFP between the case-ascertained DNM variants from Epi4K and the control groups described above.

First, we examined variants to verify whether the distribution of case-ascertained DNMs yielded significantly different scores compared to control variants. Currently, there are over 100 different *in silico* predictors available. Nevertheless, for the purpose of this study, we selected a subset available in Variant Effect Predictor (VEP) and through dbNSFP. This set provides a mixture of scores created using physicochemical properties, standing variation in the human population, and combined approaches through machine learning. Next, we utilised the provided rank scores for each metric, where we converted each score to a percentile based on all scored positions within the dataset.

**Figure 2:**
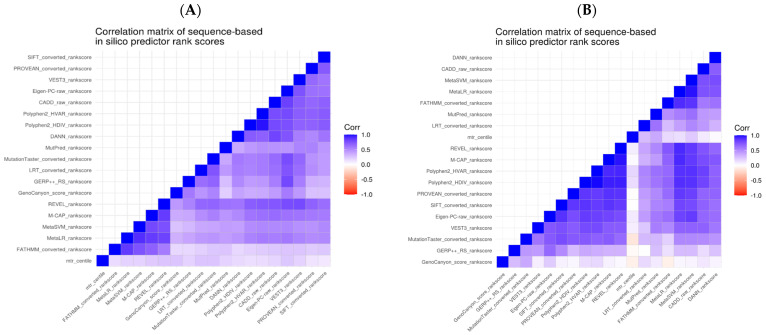
Correlation plot of *in silico* predictor scores for complete cases of variants. (**A**) Correlation of scores for all Epi4K variants across all genes. (**B**) Correlation of scores for Epi4K variants within the 34 DEE genes.

Owing to the small number of control *de novo* variants observed within the 34 DEE genes, we compared the *in silico* predictions for these as a combined set. We examined Spearman’s correlation between the predictive tools’ scores pairwise (Figure 2). Given our analysis, we could observe that the correlations were higher for the subset of variants within the DEE genes. However, overall, we noticed a correlation in the scores across all genes, especially those derived from similar properties. 

**Figure 3:**
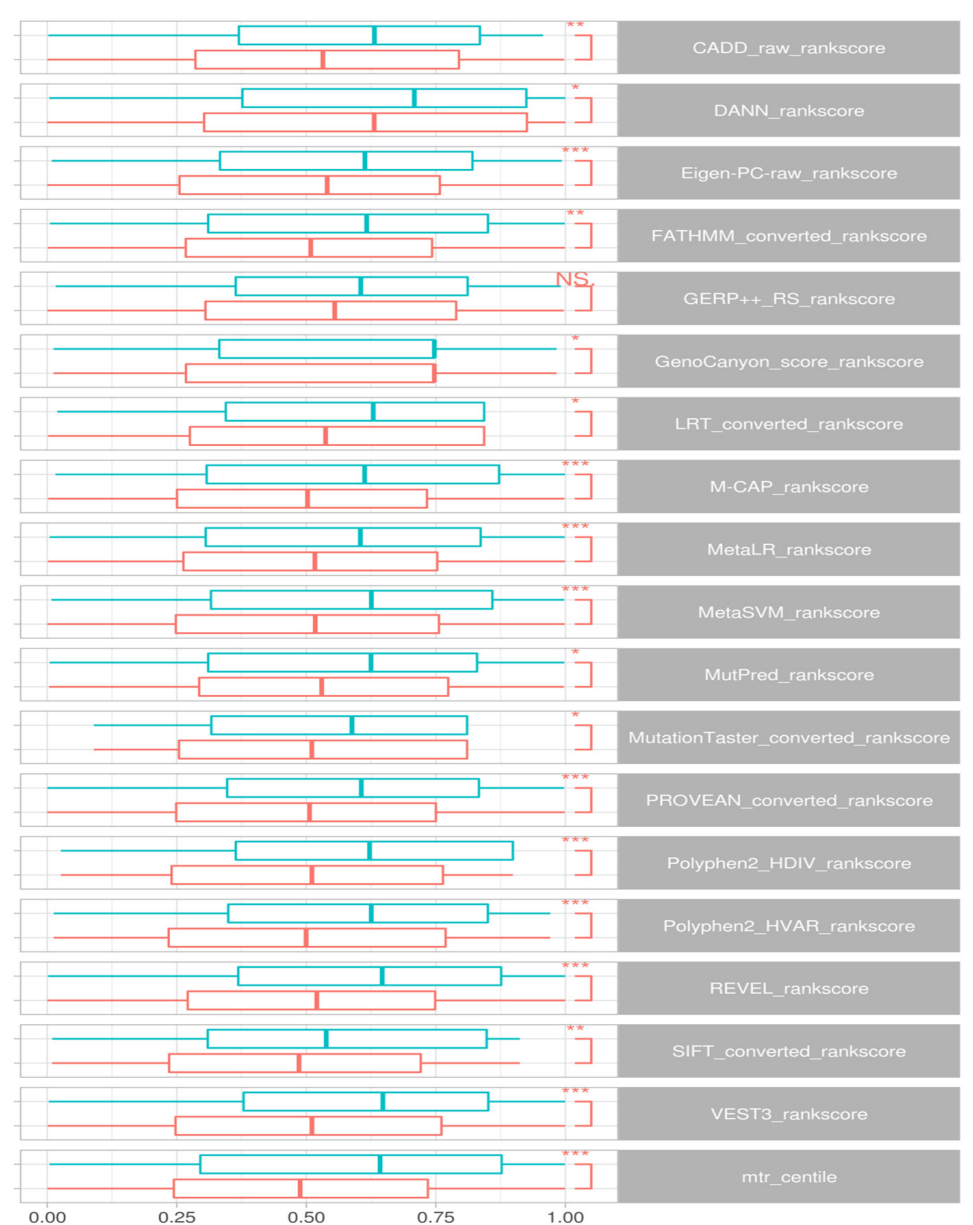
Comparison of *in silico* predictors of deleteriousness. Boxplots display rank score distributions for *de novo* missense variants across all genes, comparing case-ascertained variants (salmon) with combined variants from control groups 1 and 2 (blue). Significance was denoted by Wilcox signed rank tests, with NS not significant; * *p* < 0.05; ** *p* < 0.01; *** *p* < 0.001.

Wilcox signed rank tests comparing epilepsy DNMs to control groups identified a significant difference in rank scores for CADD (*p* < 0.008), DANN (0.03), Eigen-PC-raw (0.0008), FATHMM (*p* < 0.002), GenoCanyon (*p* < 0.02), LRT (*p* < 0.01), M-CAP (*p* < 3.8 × 10^−5^), MetaLR (*p* < 0.0004), MetaSVM (*p* < 0.0003), MutPred (*p* < 0.02), MutationTaster (*p* < 0.01), PROVEAN (*p* < 5.3 × 10^−5^), Polyphen2-HDIV (*p* < 2.9 × 10^−5^), REVEL (*p* < 1.2 × 10^−6^), SIFT (*p* < 0.002) and VEST3 (*p* < 3.8 × 10^−6^) and MTR (*p* < 1.3 × 10^−6^). We also noted that scores in GERP++ RS differ but do not achieve significance (*p* < 0.06). Figure 3 presents the distributions for these scores.

Examining the variants specific to the DEE genes, we could verify similar separation between case-ascertained and control variants for CADD (*p* < 0.005), DANN (*p* < 0.02), Eigen-PC-raw (*p* < 0.003), LRT (*p* < 0.007), M-CAP (*p* < 0.05), MetaLR (*p* < 0.01), MutationTaster (*p* < 0.0001), PROVEAN (*p* < 0.01), Polyphen2-HDIV (*p* < 5.5 × 10^−5^), REVEL (*p* < 0.009), SIFT (*p* < 0.01) and VEST3 (*p* < 0.004). Nevertheless, we could not find statistical significance for FATHMM (*p* < 0.08), GERP++ RS (*p* < 0.12), GenoCanyon (*p* < 0.11), MetaSVM (*p* < 0.15), and MutPred (*p* < 0.06). 

## 3. Discussion

*In silico* predictors of deleteriousness continue to show great utility in identifying likely pathogenic variants, with key relevance to developmental and epileptic encephalopathies. In this scenario, different studies often observe the attribution of disease to a single variant. As a result of the conceptualisation of new approaches and the availability of novel datasets, our repertoire of tools continues to expand, possibly meaning new discoveries for each variant.

Difficulties arise when researchers design tools based on the use of different human reference genomes and different transcript versions. For example, the Variant Effect Predictor and ANNOVAR are extremely useful tools in this regard. However, issues are inevitable as each tool relies on different transcripts while building and updating their respective predictors.

Given the severity and early onset of the disease, intolerance-based predictors and conservation are highly effective and valuable predictors. As also shown in a previous study of ClinVar variants, we have observed this to be the case in genes with known dominant and recessive patterns of inheritance [11], with a greater enrichment of pathogenic variants in dominant genes. Of the 34 DEE genes under investigation, 30 are within the top 10% of the most intolerant genes, indicating that variants arising within these genes in most regions (and not just the known domains) are likely deleterious. Despite the overall strong intolerance of these genes, we still see significant differences in the MTR predictions between the case and control *de novo* variants.

Using the spatial-based MTR3D, where a successful alignment between sequence and structure was available and a score could be derived, we identified 16 of the 17 case-ascertained *de novo* variants residing within intolerantly scored regions. However, due to the few control variants residing within the 34 DEE genes and with none located in a region with a valid MTR3D score, it is challenging to provide conclusions for this result. Similarly, this remains a challenge for many structure-based predictors that rely on the availability of a resolved or homology-modelled protein structure.

Nevertheless, we see great potential in their utility as an additional tool for variant prioritization, where we can calculate these scores and run such predictors. It is clear that the accuracy of predictive tools as a whole continues to improve. Surprisingly, we observed that *de novo* missense variants within DEE genes are more likely to be deleterious than control *de novo* missense variants.

These results confirm the utility of *in silico* predictors for prioritising variants in DEE and suggest that additional genes are of interest to further our understanding of the disease.

## 4. Material and Methods

### 4.1. Study Subjects and Sequencing Procedures

We evaluated three epileptic encephalopathy cohorts for this study from the Epi4K *de novo* mutations study: (1) Epilepsy Phenome/Genome Project cohort 1 (*n* = 264 trios), (2) Epilepsy Phenome/Genome Project cohort 2 (*n* = 73 trios), and (3) EuroEPINOMICS-RES cohort (*n* = 19 trios). In addition, we obtained informed consent from the parents or legal guardians of each participant, and we received approval for studies from the local ethics committees of each participating centre. Epi4K publication [3] provides further information.

We also included missense variants from the Epi25K whole-exome sequencing variant-level summary for analysis, filtered to only developmental and epileptic encephalopathy patients (*n* = 1021), thus removing samples from genetic generalised epilepsy (*n* = 3108) and non-acquired focal epilepsy (*n* = 3597). We downloaded this dataset from the Epi25 WES Browser, and it is publicly available pre-filtered for missense variants with missense badness, PolyPhen-2, and constraint (MPC) ≥ 2, which included both *de novo* and non-*de novo* variants.

We used the DiscovEHR whole-exome sequencing dataset to compare with the epilepsy cohorts [12], which included exomes from 50,000 individuals.

### 4.2. Missense Variant Annotation

We annotated Epi4K *de novo* variants using the Variant Effect Predictor (version 101) [13] and the dbNSFP 3.5a plugin [14] to include a range of predictors of deleteriousness based on physicochemical properties, conservation, and combined approaches. We also had dbNSFP’s rank scores, where we ranked each score from 0–1 based on its percentile across all scored coding positions within the dbNSFP dataset. We consider scores closer to 1 more likely to be severe. Following annotation, we selected unique variants classified as missense for subsequent analysis (case-ascertained variants: N = 276; control group: N = 454). 

We applied the same annotation procedure to the Epi25K missense variants (N = 1,082,844) and the DiscovEHR control population variants (N = 2,134,301). Similarly, we filtered these sets to only those with missense consequences.

We further annotated variants with MTR, MTR3D, and MTRX scores to explore regional intolerance to missense variation at each variant’s position. MTR3D and MTRX scores are available for a subset of epilepsy-related genes, depending on the availability of tertiary protein structures.

We selected a subset of the Epi4K *de novo* missense variants where these reside within 34 genes with evidence of being implicated in developmental and epileptic encephalopathies (DEE) as described by He et al. (2019) [6].

Moreover, we annotated ClinVar variants using the same procedure described above and filtered to those predicted to be missense and benign, likely benign, pathogenic, or likely pathogenic, omitting those with unknown significance [15]. To examine the distribution of pathogenic variants within epilepsy genes, we subset these to the previously described 34 epileptic encephalopathy genes. To ensure these are mutually exclusive for test sets, we filtered variants to exclude 16 variants that overlapped with the case-ascertained *de novo* variants.

### 4.3. Calculating Conservation

We utilised the toolset by Capra and Singh (2007) [16] to estimate sequence conservation based on Jensen-Shannon divergence from a multiple sequence alignment. We could locate some positions where variants are, but we could not measure the conservation due to gaps in aligned sequences, lowering confidence in the results. 

### 4.4. Annotating Variants with MTR3D Scores

As the MTR3D predicted scores include mappings between sequence protein positions and structural residue numbers, we used these to map the Epi4K *de novo* variants, Epi25K variants, and ClinVar pathogenic and benign variants to protein structures. We preferentially employed an experimentally resolved structure from the RCSB Protein Data Bank, where MTR3D mappings were available. Otherwise, we utilised a homology-modelled tertiary protein structure from the SWISS-MODEL database. When we could map multiple structures where we could identify a variant, we selected the structure with the highest proportion of matching sequence and structural positions. This selection varies due to partial experimental structures representing only certain regions and domains of a gene and residues that may be different due to different transcripts or substitutions to assist in the stability of the structure’s creation. For instance, we used multiple structures for genes where partial structures best fit different variants (see Supplementary Table S2).

## 5. Conclusions

Overall, we noted significant differences between case-ascertained and control variants in the majority of commonly used predictors of deleteriousness, as well as the MTR, MTR3D, and MTRX. We further show that the inclusion of structure-based predictors of intolerance improves upon previous attempts to demonstrate enrichment within epilepsy genes. These results highlight the importance of considering protein 3D structural information in the characterization of novel variants and as a key link between protein sequence and phenotype.

## Data Availability

All data used in this study is freely available in publicly accessible repositories. Data information is also provided in Supplementary Materials.

## Supplementary Materials

The following supporting information can be downloaded at: https://www.mdpi.com/article/10.3390/ijms24065114/s1.
